# Association of Health Literacy With Medication Adherence Mediated by Cognitive Function Among the Community-Based Elders With Chronic Disease in Beijing of China

**DOI:** 10.3389/fpubh.2022.824778

**Published:** 2022-04-26

**Authors:** Qiaoling Jia, Haiyan Wang, Li Wang, Yanhong Wang

**Affiliations:** Department of Epidemiology and Biostatistics, Institute of Basic Medical Sciences, Academy of Medical Sciences, School of Basic Medicine, Peking Union Medical College, Beijing, China

**Keywords:** health literacy, medication adherence, cognitive function, elder, chronic disease

## Abstract

**Background:**

Although health literacy was considered to play a crucial role in non-communicable chronic disease (NCD) prevention and control, the relationship of health literacy and medication adherence has rarely given attention among older adult Chinese population in previous studies, especially considered that they might be with cognitive impairment.

**Purpose:**

This study aimed to investigate the association between health literacy and medication adherence and mediation by cognitive ability among community-based older adults with chronic disease in Beijing of China.

**Methods:**

The older adults aged 60 years old or over were recruited in a cross-sectional survey conducted in Beijing of China by using multistage, stratified sampling method. Of those, the participants with chronic disease and need to take long-term medicine were included in our study. The information about sociodemographic characteristics, health literacy, cognition ability, and medication adherence was collected by the questionnaire. The univariate and multiple logistic regression analysis were used to measure the association of health literacy and adherence medication, and mediate effect by cognitive ability.

**Results:**

The total of 4,166 older adult populations (average age: 70.61 ± 7.38 years) was included in this study, 1,395 participants (33.49%) were non-adherence, 1,983 participants (47.60%) had two chronic conditions or more, and 1,459 participants (35.02%) screened as cognitive impairment. The health literacy was negatively associated with medication adherence. The lower total scores of health literacy were found with a high risk of non-adherence [*p* < 0.01, adjusted odds ratio (*OR*) = 0.988 per one point increase, 95% *CI*: 0.982–0.993] controlling other covariates. However, their association tended to be weakened or even disappeared among the older adults with cognitive impairment compared with the populations with normal cognitive.

**Conclusion:**

Improving health literacy might be a public health strategy to increase the medication adherence of older adults, but need to first identify the potential target population based on their cognitive ability.

## Introduction

The older adults tends to suffer from chronic diseases. According to the surveillance data of chronic diseases, nearly 75% of the population aged 60 years and over had at least one chronic disease in China (1). Usually, most of the chronic diseases need long-term multiple medicines to improve symptoms or delay the progression of disease. Adherence is a passive behavior to follow treatment recommendations prescribed by their clinicians or healthcare providers (2). It was indicated that adherence has positive and significant effects on treatment outcomes and poor adherence could reduce the effectiveness of treatment and cause more economic losses (2, 3). Previous studies reported that about 5.1~65.8% older adult patients of chronic diseases tend to have poor adherence to their medications in China, but varied by the types of chronic diseases or tools of adherence assessments (4–6). The interventions to improve the adherence were considered to make a far greater impact on the health than any improvement in specific medical treatments (2).

However, adherence was driven by many factors and these factors varied widely across the race of population, diseases, treatment regiments, and so on (7–9). Clearly and appropriately understanding the health information might be one of the essential core elements for patients to make health decisions that were closely related to adherence behaviors (9). Health literacy was defined as an ability to obtain, process, communicate, and understand the basic health information and services needed to make appropriate health decisions, which was considered to be one of the most promising and cost-effective approaches to overcome the non-communicable chronic disease (NCD) challenges (10). When patients were adequately informed and understand clearly what they were asked to do, they could actively participate in health decisions, which would help to improve their adherence to regimens (9). Therefore, improving health literacy might be an effective education and prevention strategy to improve treatment adherence. Until now, several studies had explored the association between health literacy and adherence among patients recruited from hospitals or primary health centers, but their findings were inconsistent (11–16) and their association were still ambiguity. Although health literacy was considered as a crucial role in NCD prevention and control in China, the relationship of health literacy and medication adherence has rarely given attention in the older adult Chinese population in previous studies.

Cognitive ability, which changed with aging, involved the abilities to reason, plan, solve problems, think abstractly, comprehend complex ideas, learn quickly, and learn from experience (17). Moreover, the older adults might experience subtle cognitive changes associated with aging, even those who might do not suffer from dementia or mild cognitive impairment (18). The lower cognitive function was found to be associated with poorer medicine adherence in healthy older adults (19). The study by Cho et al. reported that the decline in cognitive function worsened medicine adherence among the hypertensive patients (20). Previous studies reported that health literacy was significantly correlated with cognitive function (21–23). A cohort study by Wilson et al. found that the higher health literacy skills at baseline reduced the rate of cognitive decline in older adults (24). All of these suggested that cognitive function might be the important mediator of relationship between health literacy and medicine adherence. However, there is a lack of evidence till now, especially among the community-based population.

According to the results from the National Health Literacy Surveillance among the residents aged 15–69 years old, the health literacy among the Chinese population was still at a low level and had disparities between regions and groups (25). For the older adults 70 years old or above, the level of health literacy was still unknown in China, but might be even worse because of their low levels of education and high proportion of cognitive impairment. In this case, it was interested to know whether improving the health literacy was still an effective approach on medication adherence among the Chinese older population, and how the cognitive function mediated this effect of health literacy on adherence among the older adults in China.

Based on the above, this study aimed to investigate the association between health literacy and medication adherence among the community-based older adults with chronic disease in China. We hypothesized that the health literacy would be associated with the adherence of taking medicine, but its effect mediated by cognitive abilities.

## Materials and Methods

### Sample and Setting

Data for this analysis came from the cross-sectional health survey. The survey was conducted in December 2020 and used a multistage, stratified sampling method to select a representative sample of community-based population aged 60 years or older in Beijing of China. The sampling process was stratified according to geographic regions and development status. At first, three central districts (such as DC, FT, and SJS) and five outer districts (such as CP, HR, MY, DX, and SY) were selected randomly among 16 districts. Then, total 100 street districts and 28 rural villages were randomly selected as study sites. About 48 persons in each study site were recruited to participate in the health survey, who were 60 years or older, lived in the current residence for at least 6 months and without mental illness, deaf blindness, or long-term bedridden. The study was approved by the institutional review board of the Institute of Basic Medical Sciences, Chinese Academy of Medical Sciences (Project No. 064-2020). Written informed consent was obtained from each participant before data collection. A total of 6,160 persons participated in this survey, in which 5,829 (nearly 95% of all) were valid samples and 331 persons were eliminated for unqualified questionnaire with a lot of missing information or <60 years old. Among those, 4,166 participants who had self-reported at least one of the chronic diseases and needed to take long-term medicine were included in this analysis.

Self-completed questionnaires were used in this survey. For the older adults who were unable to complete the questionnaire by themselves owing to impaired vision, limited reading ability, or other such reasons, face-to-face inquiry by the staff from primary health center was used to collect the information. In that situation, the staffs were trained to complete the questions in a neutral fashion on the behalf of participants, and not to explain anything or help to answer questions. About 43% participants completed the questionnaire by themselves in our study.

### Measures

The information about sociodemographic characteristics (such as age, gender, ethnicity, educational attainment, married status, medical cost last year, and medical payment), health literacy, cognition, and medication adherence were collected in the survey.

The Chinese version of the eight-item Morisky Medication Adherence Scale (MMAS-8) was used to assess the medication adherence (26–28). This scale included 7 items with “yes” or “no” answers and 1 item scored by an ordinal scale from 0 to 4. Items 5 and 8 of the questionnaire were transformed in accordance with the scoring algorithm, and all items were combined into a total score, ranging from 0 to 8 points. According to the scores, the older adult populations were divided into groups with low (<6 points), moderate (at least 6 points, but <8 points), and high (8 points) adherence to drug regimen. In our analysis, the low adherence was defined as non-adherence, moderate or high was defined as adherence. The Cronbach's α coefficient for the MMAS-8 was 0.76 in our study.

Health literacy was assessed by the Chinese Resident Health Literacy Scale (29). This scale contained 56 items with total scores ranging from 0 to 66 points and three dimensions: (1) knowledges and attitudes (28 points, Cronbach's α coefficient: 0.76); (2) behavior and lifestyle (22 points, Cronbach's α coefficient: 0.72); and (3) health-related skills (16 points, Cronbach's α coefficient: 0.66). The questions covered six aspects: scientific views of health (11 points); infectious diseases (7 points); chronic diseases (12 points); safety and first aid (14 points); medical care (14 points); and health information (8 points). The total scores reflected the health literacy level and the higher scores indicated higher health literacy, and vice versa.

The Ascertain Dementia 8 (AD8) questionnaire was used for screening the cognitive impairment in this study (30). AD8 is a brief informant-based measure that had only eight questions, such as domains of judgments, hobby/activity levels, repetitive conversations, learning abilities, memory in relation to date/appointments, finance, and daily thought processes. The AD8 had a good diagnostic accuracy in discriminating the cognitive impairment from normal cognition (31). The person with an AD8 score ≥2 was suspected to have cognitive impairment and need to be further definite diagnosis (30, 31). Cronbach's α coefficient of AD8 was 0.87 in our study.

### Statistical Analysis

Descriptive statistics were calculated for all participant characteristics. Chi-square test, *t*-test, and ANOVA tests were conducted to compare demographic characteristics and health literacy scores between non-adherence and adherence. Taking non-adherence as the dependent variable, bivariate logistic regression analyses were performed to derive univariate, adjusted odds ratios (*OR*) and their respective 95% confidence intervals (*CI*s). Using the scores of health literacy, scores of knowledges and attitudes, scores of behavior and lifestyle, scores of health-related skills, scores of scientific views of health, scores of infectious diseases, scores of chronic diseases, scores of safety and first aid, scores of medical care, and scores of health information, respectively, as independent variable, the multiple logistic regression model was fit to estimate multivariate *OR*, controlling for developed status (urban or rural), age, gender, education attainment, married status, smoke, one or more of chronic disease, medical cost for last year, self-completed questionnaire, and cognitive impairment. The population was divided into cognitive impairment (AD8 scores at least 2 points) and normal cognitive, then in each subgroup, we also explored the association between health literacy and medication adherence after controlling other covariates. Furthermore, the scores were divided, respectively into four quartiles, e.g., Q1 (the lowest 25%), Q2 (50% or less), Q3 (75% or less), and Q4 (the highest 75%) to investigate the effect of modification by cognition on the association between health literacy and medicine adherence. The Cronbach's coefficient was calculated to examine the internal reliability of the scales or subscales used in this study. Statistical significance was accepted at *p* < 0.05 and all analyses were conducted using SAS 9.4 software (SAS Institute, Cary, NC, USA).

## Results

A total of 4,166 older adult populations with self-reported one or more chronic conditions were included in this study, and average age was 70.61 ± 7.38 years, ranging from 60 to 100 years old. Of those, 1,395 participants (33.49%) were identified as non-adherence (scores of MMAS-8 <6 points), and 1,983 participants (47.60%) self-reported to have at least two chronic conditions or more, and 1,459 participants (35.02%) might be cognitive impairment screened by AD8 (scores of AD8 ≥ 2 points). The characteristics of the older adults are shown in Table 1.

**Table 1:** Characteristics of study participants and comparison between adherence and non-adherence.

	**Total (*N* = 4,166) *n* (%)**	**Adherence (*N*= 2 771) *n* (%)**	**Non-adherence (*N* = 1,395) *n* (%)**	*P*-value^*^	**Univariate OR (95%CI)#**
Type of residence					
Urban	3,280 (78.73%)	2,261 (68.93%)	1,019 (31.07%)	<0.001	Reference
Rural	886 (21.27%)	510 (57.56%)	376 (42.44%)		1.636 (1.405–1.905)
Gender					
Male	2,025 (49.47%)	1,382 (68.25%)	643 (31.75%)	0.035	Reference
Female	2,068 (50.53%)	1,347 (65.14%)	721 (34.86%)		1.150 (1.010–1.310)
Subgroups of age					
60–69	1,988 (47.72%)	1,347 (67.76%)	641 (32.24%)	0.008	Reference
70–79	1,608 (38.6%)	1,077 (66.98%)	531 (33.02%)		1.036 (0.901–1.192)
≥80 or over	570 (13.68%)	347 (60.88%)	223 (39.12%)		1.350 (1.114–1.638)
Ethnicity					
Han	3,992 (96.05%)	2,663 (66.71%)	1,329 (33.29%)	0.173	Reference
Other	164 (3.95%)	101 (61.59%)	63 (38.41%)		1.250 (0.906–1.724)
Education					
Illiterate or primary school	1,135 (27.24%)	633 (55.77%)	502 (44.23%)	<0.001	Reference
Junior school	1,552 (37.25%)	1,085 (69.91%)	467 (30.09%)		0.543 (0.463–0.637)
High school, college, or graduate school	1,479 (35.50%)	1,053 (71.20%)	426 (28.80%)		0.510 (0.434–0.600)
Marital status					
Unmarried, divorced, widow	735 (17.92%)	455 (61.90%)	280 (38.10%)	0.002	Reference
Married	3,367 (82.08%)	2,284 (67.83%)	1,083 (32.17%)		0.771 (0.653–0.909)
Smoking condition					
Never smoking	2,670 (65.17%)	1,801 (67.45%)	869 (32.55%)	0.183	Reference
Smoking	790 (19.28%)	509 (64.43%)	281 (35.57%)		1.144 (0.969–1.352)
Smoked, but quit now	637 (15.55%)	413 (64.84%)	224 (35.16%)		1.124 (0.937–1.348)
Healthcare costs in last year					
<5000 Yuan	2,563 (61.52%)	1,748 (68.2%)	815 (31.80%)	0.004	Reference
≥5000 Yuan	1,603 (38.48%)	1,023 (63.82%)	580 (36.18%)		1.216 (1.066–1.387)
Medical insurance					
No	46 (1.11%)	26 (56.52%)	20 (43.48%)	0.153	Reference
Yes	4,086 (98.89%)	2,718 (66.52%)	1,368 (33.48%)		0.654 (0.364–1.176)
Suffering from chronic diseases					
Only one	2,183 (52.40%)	1,501 (68.76%)	682 (31.24%)	0.001	Reference
Multiple (2 or more)	1983 (47.60%)	1270 (64.04%)	713 (35.96%)		1.236 (1.086–1.406)
Cognitive ability					
Cognitive normal	2,707 (64.98%)	2,092 (77.28%)	615 (22.72%)	<0.001	Reference
Cognitive impairment	1,459 (35.02%)	679 (46.54%)	780 (53.46%)		3.908 (3.409–4.480)
Questionnaire filling method					
Self-completed	1,807 (43.37%)	1,150 (63.64%)	657 (36.36%)	<0.001	Reference
Face-to-face inquiry	2,359 (56.63%)	1,621 (68.72%)	738 (31.28%)		0.797 (0.700–0.907)
Total scores of health literacy (Mean±SD)	39.21 ± 12.71	40.38 ± 12.35	36.88 ± 13.07	<0.001	0.979 (0.974–0.984)
cores of three dimensions (Mean ± SD)					
Knowledges and attitudes	17.24 ± 5.53	17.67 ± 5.43	16.38 ± 5.62	<0.001	0.959 (0.948–0.97)
Behavior and lifestyle	12.81 ± 4.88	13.26 ± 4.76	11.90 ± 5.01	<0.001	0.944 (0.932–0.957)
Health-related skills	9.16 ± 3.57	9.45 ± 3.48	8.60 ± 3.69	<0.001	0.936 (0.919–0.953)
Scores of covering six aspects (Mean ± SD)					
Scientific views of health	6.74 ± 2.79	6.92 ± 2.74	6.37 ± 2.86	<0.001	0.932 (0.910–0.953)
Infectious diseases	4.44 ± 1.72	4.56 ± 1.68	4.20± 1.76	<0.001	0.886 (0.853–0.920)
Chronic diseases	6.64 ± 2.95	6.85 ± 2.91	6.23 ± 2.96	<0.001	0.931 (0.911–0.952)
Safety and first aid	9.49 ± 3.39	9.82 ± 3.26	8.84 ± 3.55	<0.001	0.919 (0.902–0.937)
Medical care	7.79 ± 2.83	8.03 ± 2.78	7.31 ± 2.88	<0.001	0.914 (0.894–0.936)
Health information	4.11 ± 2.10	4.20 ± 2.08	3.92 ± 2.12	<0.001	0.938 (0.909–0.967)

* *p for chi-square test, t-test or ANOVA tests, p <0.05*.

*^#^Univariate bivariate logistic models with adherence as the dependent variable (event = “Non-adherence”)*.

*The MMAS-8 Scale, content, name, and trademarks are protected by the US copyright and trademark laws. Permission for use of the scale and its coding is required. A license agreement is available from MMAR, LLC., Donald E. Morisky, ScD, ScM, MSPH, 294 Lindura Ct., USA; donald.morisky@moriskyscale.com*.

### The Association of Health Literacy With Medication Adherence

As shown in Table 1, participants who were living in urban areas, female, older adult, educated with illiterate or Primary school, as well as unmarried, divorced, or widowed tend to have a higher risk of non-adherence. The comorbidity, higher healthcare costs in last year, and cognitive impairment were also associated with the risk of poor medication adherence.

Moreover, health literacy was negatively associated with medication adherence. The lower total scores were found among the participants with non-adherence (*p* < 0.01, crude *OR* = 0.979 per one point increase, 95% *CI*: 0.974–0.984) when not controlling for any other covariates. The similar results were found in each dimension and each covering aspect of health literacy (as shown in Table 1).

In adjusted analyses (Table 2), the total scores of health literacy also associated with medication adherence when controlling other covariates (such as, type of residence, gender, age, education, marital status, smoking condition, healthcare costs in last year, comorbidity, and self-completed questionnaire) and adjusted *OR* was 0.983 (95% *CI*: 0.977–0.988), so did the scores of each dimension, as well as scores of each covering aspect of health literacy. Furthermore, when controlling the covariate of cognitive ability, the association of health literacy and adherence was still found (except the scores of health information).

**Table 2:** Multivariable-adjusted odds ratios (*OR*s) for the association between the scores of health literacy and medication adherence.

	**Model 1^#^**	**Model 2^##^**	**Model 3^###^**
	**OR (95%CI)**	**OR (95%CI)**	**OR (95%CI)**
Total scores of health literacy	0.983 (0.978–0.989)	0.983 (0.977–0.988)	0.988 (0.982–0.993)
Cognitive impairment	N.A.	N.A.	3.464 (2.983–4.023)
Scores of three dimensions			
Knowledges and attitudes	0.971 (0.959–0.983)	0.968 (0.956–0.981)	0.979 (0.967–0.992)
Cognitive impairment (ref = Normal)	N.A.	N.A.	3.503 (3.017–4.067)
Behavior and lifestyle	0.952 (0.939–0.966)	0.953 (0.939–0.967)	0.963 (0.949–0.978)
Cognitive impairment (ref = Normal)	N.A.	N.A.	3.471 (2.989–4.030)
Health-related skills	0.952 (0.934–0.970)	0.947 (0.929–0.966)	0.964 (0.945–0.984)
Cognitive impairment (ref = Normal)	N.A.	N.A.	3.494 (3.009–4.057)
Scores of covering six aspects			
Scientific views of health	0.951 (0.928–0.974)	0.947 (0.924–0.971)	0.965 (0.940–0.99)
Cognitive impairment (ref = Normal)	N.A.	N.A.	3.529 (3.040–4.097)
Infectious diseases	0.910 (0.875–0.946)	0.917 (0.881–0.954)	0.937 (0.899–0.976)
Cognitive impairment (ref = Normal)	N.A.	N.A.	3.538 (3.049–4.106)
Chronic diseases	0.948 (0.926 - 0.969)	0.947 (0.925 - 0.969)	0.964 (0.941–0.988)
Cognitive impairment (ref = Normal)	N.A.	N.A.	3.522 (3.034–4.088)
Safety and first aid	0.936 (0.917–0.955)	0.934 (0.915–0.953)	0.953 (0.933–0.974)
Cognitive impairment (ref = Normal)	N.A.	N.A.	3.452 (2.972–4.010)
Medical care	0.928 (0.906–0.950)	0.922 (0.900–0.945)	0.941 (0.918–0.965)
Cognitive impairment (ref = Normal)	N.A.	N.A.	3.468 (2.987–4.027)
Health information	0.965 (0.935–0.996)	0.955 (0.924–0.987)	0.969 (0.936–1.003)
Cognitive impairment (ref = Normal)	N.A.	N.A.	3.569 (3.075–4.141)

# *Model 1: Bivariate logistic models those adherence was as the dependent variable (event = “Non-adherence”), and the scores of health literacy (or scores of knowledges and attitudes, or scores of behavior and lifestyle, or scores of health-related skills, or scores of scientific views of health, or scores of infectious diseases, or scores of chronic diseases, or scores of safety and first aid, or scores of medical care, or scores of health information, respectively) as independent variable, controlling for variables, such as type of residence, gender, age, and education*.

## *Model 2: Further adding variables of marital status, smoking condition, medical costs for last year, multiple chronic disease, as well as questionnaire filling method as covariates in Model 1*.

### *Model 3: Adding variable of cognitive impairment in Model 2*.

### Cognitive Condition Mediation Analysis

Then, the older adults were divided into cognitive normal and cognitive impairment according to AD8 scores. It was found the total scores of health literacy, the scores of each dimension, as well as the scores of each covering aspect were also negatively associated with non-adherence among the elderly with normal cognitive (Table 3). However, among the older adults with cognitive impairment, these associations were only found in total scores, the scores of behaviors and lifestyles, the scores of safe and first aid, as well as the scores of medical cares (Table 3).

**Table 3:** Multivariable-adjusted *OR*s for the association between health literacy and medication adherence among the participants with or without cognitive impairment.

	**Participants without cognitive impairment**	**Participants with cognitive impairment**
Total scores of health literacy	0.985 (0.977–0.993)	0.991 (0.982–0.999)
Scores of three dimensions		
Knowledges and attitudes	0.974 (0.957–0.991)	0.987 (0.967–1.007)
Behavior and lifestyle	0.958 (0.940–0.978)	0.969 (0.948–0.991)
Health-related skills	0.957 (0.931–0.984)	0.972 (0.943–1.003)
Scores of covering six aspects		
Scientific views of health	0.952 (0.919–0.986)	0.981 (0.944–1.020)
Infectious diseases	0.895 (0.846–0.946)	0.985 (0.926–1.048)
Chronic diseases	0.965 (0.934–0.996)	0.964 (0.928–1.001)
Safety and first aid	0.949 (0.922–0.977)	0.957 (0.928–0.988)
Medical cares	0.939 (0.908–0.972)	0.943 (0.907–0.980)
Health information	0.944 (0.902–0.988)	1.003 (0.950–1.059)

*Bivariate logistic models those adherence was as the dependent variable (event = “Non-adherence”), and the scores of health literacy (or scores of knowledges and attitudes, or scores of behavior and lifestyle, or scores of health-related skills, or scores of scientific views of health, or scores of infectious diseases, or scores of chronic diseases, or scores of safety and first aid, or scores of medical care, or scores of health information, respectively) as independent variable, controlling for type of residence, gender, age, education, marital status, smoking condition, medical costs for last year, multiple chronic disease, as well as questionnaire filling method*.

**Figure 1:**
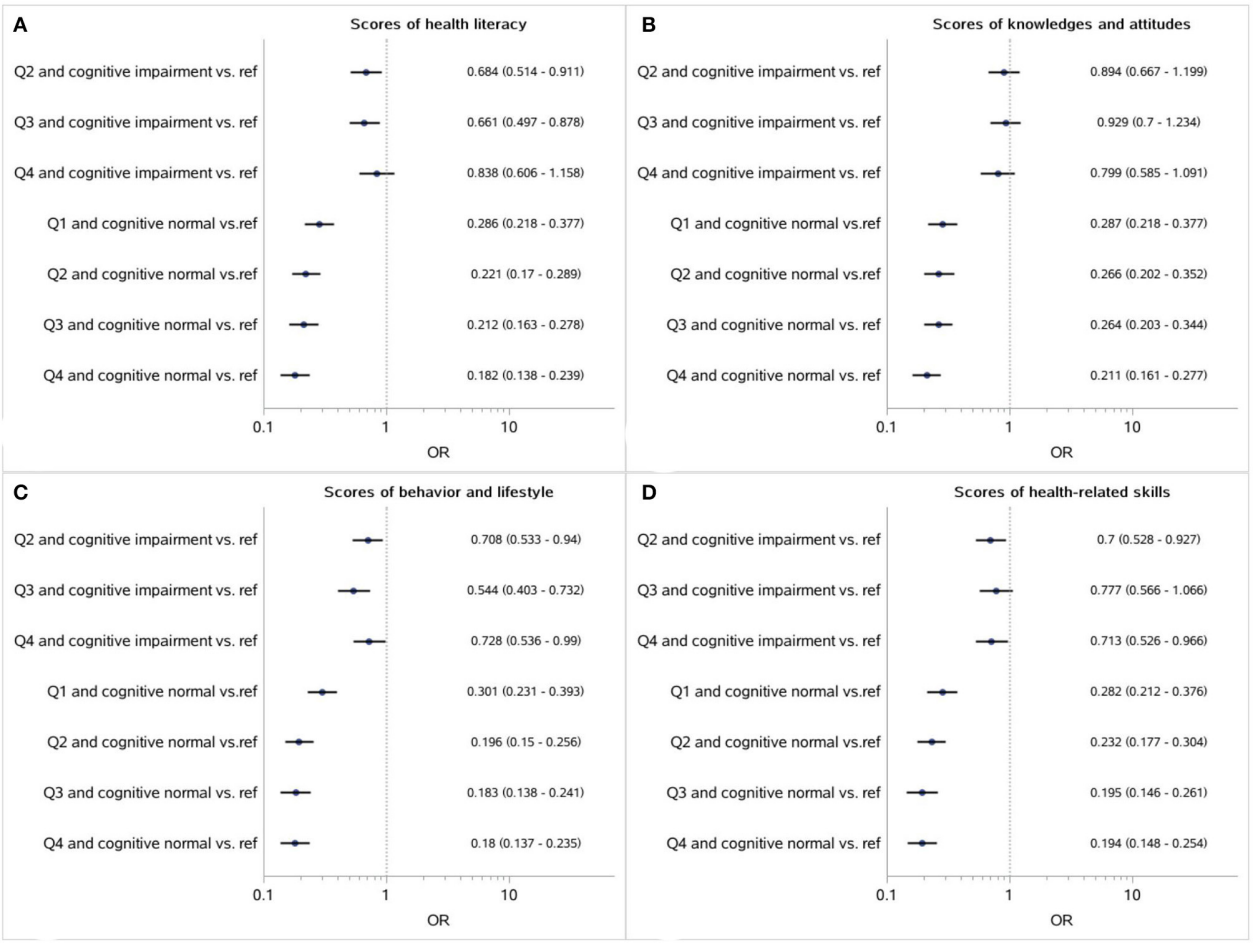
Association between the respective scores of health literacy, as well as three dimensions (**A**: Healthy literacy; **B**: Knowledges and attitudes; **C**: Behavior and lifestyle; **D**: Health-related skills) and medication adherence mediated by cognitive impairment (Reference: participants with the lowest 25% scores and cognitive impairments).

**Figure 2:**
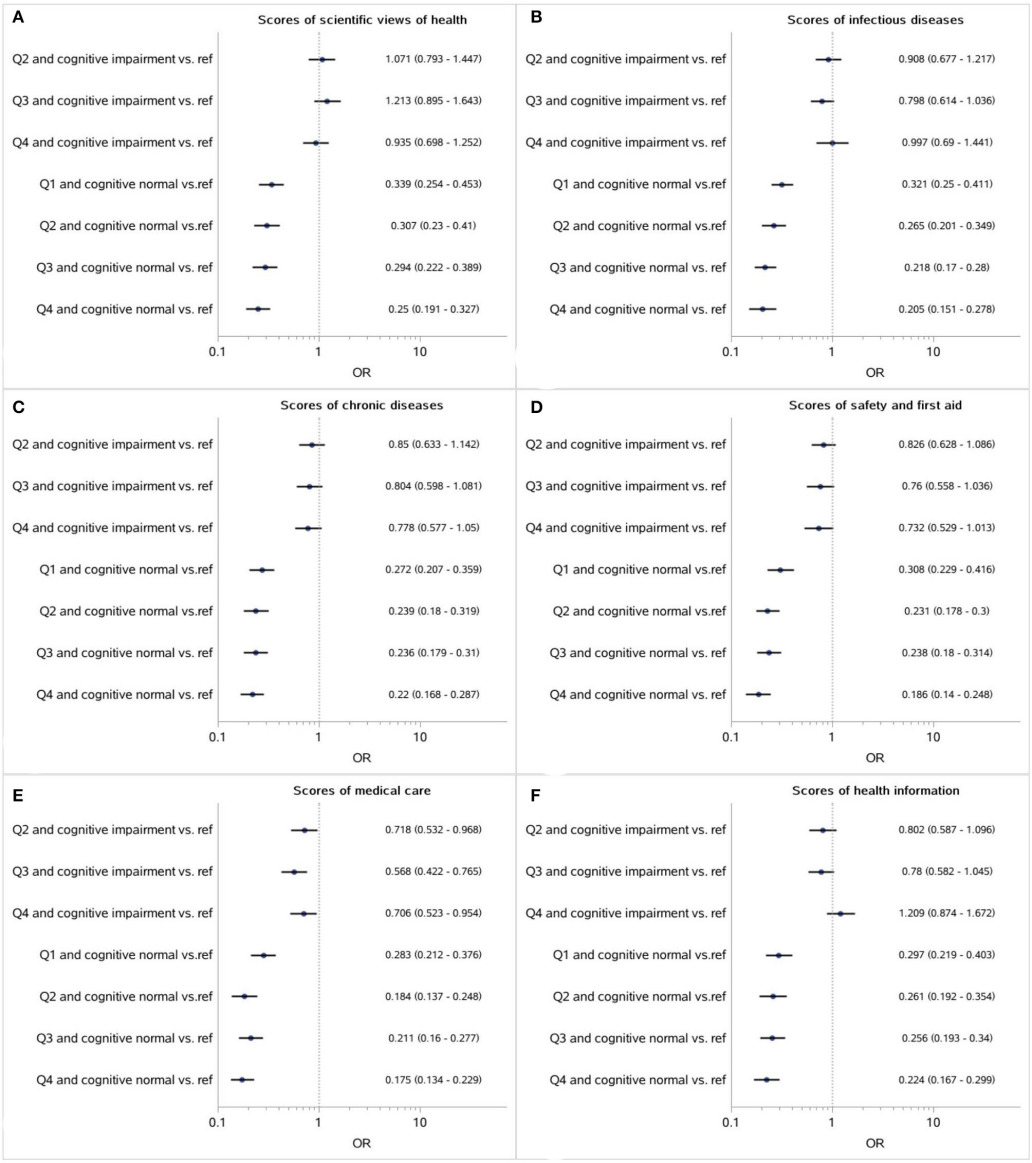
Association between the respective scores of covering six aspects (**A**: Scientific views of health; **B**: Infectious diseases; **C**: Chronic diseases; **D**: Safety and first aid; **E**: Medical care; **F**: Health information) and medication adherence mediated by cognitive impairment (Reference: participants with the lowest 25% scores and cognitive impairments).

Furthermore, according to the quantile of each score and cognitive normal/impairment, the participants were divided into eight subgroups. Compared with the subgroup with the lowest 25% scores and cognitive impairment, lower adjusted *OR* was found among the cognitive normal population in which adequate health literacy was still a positive factor of medication adherence, while the effects of health literacy on adherence were weakened or even disappeared among the older adults with cognitive impairments (as shown in Figures 1, 2).

## Discussion

This study was focused on the relation of health literacy to medication adherence on the community-based older adults with one or more chronic conditions in Beijing of China. Of these older adults, nearly one-third were identified as non-adherence to medication and the higher scores of health literacy were found to be negatively associated with the risk of non-adherence in our study, especially among the older adults with normal function. In addition, we found that the cognitive function played an important mediator and the association of health literacy with adherence tended to be weakened or even disappeared among the older adults with cognitive impairment compared with those with normal cognitive.

In a meta study, Miller synthesized both correlation and intervention studies reported that health literacy was positively associated with adherence (8). Soones et al. indicated that health literacy had a direct and an indirect effect on adherence among the older adults with asthma (16). Our results in this study were similar to the findings in these previous studies. The association of health literacy with adherence might be bidirectional in this study. On the one hand, the individuals with poor health literacy had limited ability to obtain, process, and understand the health information, so that they had difficulty in understanding instructions rightly, even prone to develop fears about the side effect and addition of medication, and ultimately resulted in decreased adherence to taking medicine. On the other hand, non-adherence was found to be the worse health outcome (2, 3), which made them to actively obtain more information and understand instructions by communicating with the healthcare provider or by other channels (e.g., internet and televisions) so that they could make appropriate health decisions if needed, which, in turn, might improve health literacy.

The association between health literacy and adherence was not only affected by reading and numeracy, but also by the abilities necessary to actively learn and apply new information and crystallized abilities (such as, background knowledge), all of them were closely related to cognitive ability (23). Our findings suggested that the cognitive ability played an important mediator in these association. For the older adults with normal cognitive, improving health literacy might still be one of public health strategies to increase the medication adherence to deal with NCDs challenge, especially for the older adults with poor health literacy. However, some of the protective effects of improving health literacy might be offset by the larger impact of cognitive impairment with aging. The findings implied that the intervention among the older adults aimed at improving the health literacy need first to identify the sensitive population or potentially valuable targets and consider the active implementation of strategies according to their cognitive ability. The action programs for the improvement of health literacy for the older adults should incorporate cognitive science, which should design the easily understandable education materials, and decompose multi-steps or complex skills into small steps to teach, encourage the older population to repeat the information in their own words. Furthermore, for the older adult individuals with cognitive impairments, the strategies focused on the improvement of cognitive function might be more effective to improve their medication adherence, such as cognitive training, behavioral therapy, or administration of cholinesterase inhibitors.

This study was based on a representative sample of the older adult residents living in Beijing. In total, 4,166 participants suffering from one or more conditions reported by themselves were included in our analysis, which accounted for 71.47% of all the participants (4,166/5,829) in this community-based cross-sectional survey. This proportion were close to the proportion of 75% NCD previously reported in the Chinese older population (1). Our study first provided the evidence of adherence to medication from the general older adults in Beijing of China. In addition, our study found the effects of health literacy on adherence mediated by cognitive function among the Chinese older adults, which rarely reported previously. However, there were still limitations in our study. First, the cognitive impairment in our study was identified by AD8 scale which was just a screen tool and not a diagnosis tool. Although there was good accuracy (sensitivity: 92%; specificity: 46%) for AD8 scale, the potential misclassification (especially those of the older adult with normal cognitive misclassified as “cognitive impairment” identified by AD8) might lead to overestimate the effects of health literacy among the participants with cognitive impairment. Second, about 56.6% of participants provided the information by face-to-face inquiry owing to impaired vision, or limited reading ability or other such reasons, which might cause information bias especially for the measurement of health literacy. Although the type of questionnaire filled were put as covariate in the multiple models in our analysis, there might be still the potential residual confounding. Third, there were no widely recognized effective tools for assessing the level of health literacy mainly focused on the older adults in China. In our study, we used Chinese Resident Health Literacy Scale which was a commonly used measure among adults of 15–69 years older, but lack of evidence for 70 years old or over. Consider that the cut-off point (55 points, 80% of 66 points) for distinguishing adequate health literacy in this scale might be more rigorous for the older adults, especially the aged older adults, we used the scores and their quantile to assess the level of health literacy in our analysis.

## Conclusion

In our study, we found that the one-third of the older adults with chronic diseases were non-adherence to the medication in Beijing of China and the association of health literacy to adherence mediated by cognitive ability. The findings suggested that improving health literacy might be one of the public health strategies to increase adherence to medication, especially for the older adults of poor health literacy. But, the priority to identify the potential target population is needed considering the cognitive ability.

## Data Availability Statement

The original data supporting the conclusions of this article will be provided by the authors, further inquiries can be directed to the corresponding author.

## Ethics Statement

The studies involving human participants were reviewed and approved by the Institutional Review Board of the Institute of Basic Medical Sciences, Chinese Academy of Medical Sciences (Project No. 064-2020). The patients/participants provided their written informed consent to participate in this study.

## Author Contributions

YW and LW participated in the design of the study and organized the training of investigators. QJ and HW participated in data collection and quality control. QJ and YW drafted the manuscript, performed the statistical analysis, and revision of the manuscript. All authors contributed to the article and approved the submitted version.

## Funding

The survey was funded by the Beijing Municipal Health Commission and the Beijing Health Economics Association. The funder had no role in the design of the study and collection, analysis, interpretation of data, or writing the manuscript.

## Conflict of Interest

The authors declare that the research was conducted in the absence of any commercial or financial relationships that could be construed as a potential conflict of interest.

## Publisher's Note

All claims expressed in this article are solely those of the authors and do not necessarily represent those of their affiliated organizations, or those of the publisher, the editors and the reviewers. Any product that may be evaluated in this article, or claim that may be made by its manufacturer, is not guaranteed or endorsed by the publisher.
